# Droplet-engineered organoids recapitulate parental tissue transcriptome with inter-organoid homogeneity and inter-tumor cell heterogeneity

**DOI:** 10.1016/j.fmre.2022.05.018

**Published:** 2022-06-03

**Authors:** Haoran Zhao, Yifan Cheng, Jiawei Li, Jiaqi Zhou, Haowei Yang, Feng Yu, Feihong Yu, Davit Khutsishvili, Zitian Wang, Shengwei Jiang, Kaixin Tan, Yi Kuang, Xinhui Xing, Shaohua Ma

**Affiliations:** a Tsinghua Shenzhen International Graduate School (SIGS), Tsinghua University, Shenzhen 518055, China; b Tsinghua-Berkeley Shenzhen Institute (TBSI), Shenzhen 518055, China; c Department of Chemical and Biological Engineering, Hong Kong University of Science and Technology, Hong Kong 999077, China; d HKUST Shenzhen Research Institute, Shenzhen 518057, China

**Keywords:** Liver organoid, Tumor, Droplet-engineered organoid, RNA-seq, Droplet-based microfluidics

## Abstract

Organoids are expected to function as effective human organ models for precision cancer studies and drug development. Currently, primary tissue-derived organoids, termed non-engineered organoids (NEOs), are produced by manual pipetting or liquid handling that compromises organoid-organoid homogeneity and organoid-tissue consistency. Droplet-based microfluidics enables automated organoid production with high organoid-organoid homogeneity, organoid-tissue consistency, and a significantly improved production spectrum. It takes advantage of droplet-encapsulation of defined populations of cells and droplet-rendered microstructures that guide cell self-organization. Herein, we studied the droplet-engineered organoids (DEOs), derived from mouse liver tissues and human liver tumors, by using transcriptional analysis and cellular deconvolution on bulk RNA-seq data. The characteristics of DEOs are compared with the parental liver tissues (or tumors) and NEOs. The DEOs are proven higher reproducibility and consistency with the parental tissues, have a high production spectrum and shortened modeling time, and possess inter-organoid homogeneity and inter-tumor cell heterogeneity.

## Graphical abstract

Droplet-engineered organoids (DEOs), compared with non-engineered organoids (NEOs), recapitulate parental tissue transcriptome better with inter-organoid homogeneity and inter-tumor cell heterogeneity. In this paper, side-by-side comparisions between DEOs and NEOs from both mouse normal tissue and human liver cancer sample have been performed to verify the superior consistency and similarty of DEOs.

**Figure:**
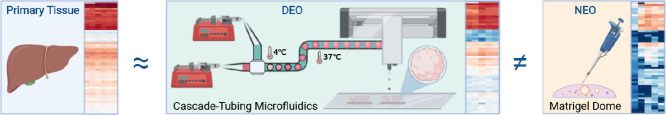

## 1 Introduction

An organoid aims to recapitulate its parental organ's structure and function on a smaller scale. Its potential capacity to mimic the response of a parental organ toward a drug endows its promises in precision medicine and drug development. An organoid can be derived from different cell sources, including pluripotent stem cells (PSCs) [1], adult stem cells (ASCs) [2], and dissociated tissues [3]. Accordingly, an organoid can be classified as a single-cell-derived organoid or dissociated tissue-derived organoid [4]. Tissue-derived organoids (or patient-derived organoids) are proven extremely competitive in predicting drug responses of their parental organs. For example, Vlachogiannis et al. reported 100% sensitivity, 93% specificity, 88% positive predictive value, and 100% negative predictive value in predicting treatment response of metastatic gastrointestinal cancers by using patient-derived tumor organoids [5]. Prediction of personalized responses toward chemoradiation [6] and DNA repair inhibitors [7] have also been reported with high accuracy.

Despite the aforementioned successes in laboratories, the current organoid technologies face many challenges toward translation, one of which is batch-to-batch and organoid-to-organoid inconsistency, i.e. irreproducibility. For example, manual dome deposition of cell-in-Matrigel suspension cannot control the initial cell distribution that is critical for organoid colony production. It may result in low rates of organoid growth or poor recapitulation of parental tissue features [8]. Moreover, manual operation, as reported in most studies, raises labor costs and cell consumption, and reduces organoid reproducibility.

Liver disease is among the most prevalent diseases in humans. Advanced modeling protocols for the liver with both healthy and pathological features are established to study liver development [9], regeneration [10], metabolism [11], and cancer [12,13]. For example, human iPSCs-derived liver organoids recapitulate hepatobiliary organogenesis *in vitro* [9] and human primary liver cancer-derived organoids are developed for disease modeling and drug screening [13]. However, primary tissue-derived liver organoids by manual operation are below satisfactory because of their poor reproducibility and low transcriptomic similarity with their parental tissue.

Reproducible organoid fabrication must satisfy the needs of lacking a precise definition on the spatial distribution of source cells, compromising organoid complexity and microenvironment recovery. Reproducible organoid fabrication has been suggested to proceed via different principles. Among those, patient-derived colorectal cancer (CRC) organoids are constructed in arrays by in-cavity aggregation of pluripotent stem cells, in which the expression of genes -involved in the production of ECM components and the surrounding microenvironment were exclusively upregulated in tumoroids attained on the micro-engineered hydrogel substrates [14]. Liver and lung cancer-on-a-chip were constructed in microfluidic chips composed of multi-channel concentration gradients, precision micro-nano structures, and hydrogel lamination [15,16]. The microfluidics droplet-engineering method [17] was reported to produce primary tissue-derived organoids, including mouse liver organoids and human liver tumor organoids in a highly reproducible manner. It consumes only around a thousand cells to construct an organoid of over 400 μm across within 1 week, and a biopsy piece allows the production of nearly 100 organoids. All the aforementioned methods achieved reproducible fabrication by precisely controlling the cell distribution, either in 3D aggregation, 2D lamination, or droplet encapsulation. Notably, the droplet-engineering method is by far one of the few reported methods to process primary tissue-derived organoids with high reproducibility.

Even though the droplet-engineering method has proved its efficacy in drug evaluation for personalized medicine, there has been no fundamental study to understand its capacity in recapitulating the function, transcription, and cell composition of the parental tissues, as well as the assessment of its advantage against the manual products. In this work, we compared droplet-engineered organoids (DEOs) and non-engineered organoids (NEOs), in reference to mouse liver tissues and human liver tumors, from where the organoids were derived. Among them, the NEOs refer to the organoids grown from the manual deposition of suspended cells in Matrigel domes or sheets [10].

## 2 Experimental model and subject details

### 2.1 Mice

All animal experiments were approved by the Animal Ethical Committee of Tsinghua University Shenzhen International Graduate School and performed in accordance with the Guidelines for the Care and Use of Laboratory Animals. Adult male BALB/c mice were purchased from Guangdong Medical Laboratory Animal Center and maintained in the Tsinghua University Laboratory Animal Center.

### 2.2 Human Specimens

The collection of patient data and liver tumor tissue has been performed according to the guidelines of the Second People's Hospital of Shenzhen Ethics Committees following both the national and the local laws of China. All participants provided written informed consent prior to the procedure.

### 2.3 Method details

#### 2.3.1 Mouse tissue processing

BALB/c mice (male, 6–8-weeks old) were euthanized by excessive CO_2_. Then, the livers were isolated and cut into small pieces (1–3 mm^3^ for each piece). After being washed with cold PBS (1 ×) solution three times and supplemented with 2.0% penicillin-streptomycin (vol/vol, GIBCO), the tissues were digested by 1.0 mg/mL collagenase type I (Sigma-Aldrich) and 10% fetal bovine serum (FBS) (vol/vol, Invigentech) on an orbital shaker at 37 °C and incubated for 1–2 h. After digestion, the tissues were sheared by 10 mL plastic pipettes. The suspension was passed through a 100 μm filter (Falcon) and centrifuged at 1000 rpm for 5 min. The pellet was resuspended in 5 mL red blood cell lysis buffer (Roche) for 5 min at room temperature. Afterward, the suspension was recentrifuged at 1000 rpm for 5 min, and the pellet was resuspended in DMEM/F12 medium. The cell number was counted for organoid fabrication.

#### 2.3.2 Human liver tumor processing

The remnant tissue samples from surgical resections after clinicopathological diagnosis were utilized for research. The specimens were immersed in an organic preservation solution (AQIX, UK) and transported from the hospital to the laboratory at 4 °C. The specimens were used to extract cells for fabricating tumor organoids and extract RNA for transcriptome analysis.

Tumor tissues were washed three times with cold PBS (1 ×) solution with 2.0% penicillin-streptomycin (vol/vol, GIBCO) and then cut into small pieces. The tissues were then digested by 1.0 mg/mL collagenase type I (Sigma-Aldrich) and 2.0% fetal bovine serum (FBS) (vol/vol, Invigentech) on an orbital shaker at 37 °C and incubated for 1–2 h. After digestion, the tissues were sheared by 10 mL plastic pipettes. The suspension was passed through a 100 μm filter (Falcon) and centrifuged at 1000 rpm for 5 min. The pellet was then resuspended in 5 mL red blood cell lysis buffer (Roche) for 5 min at room temperature. Afterward, the suspension was re-centrifuged at 1000 rpm for 5 min, and the pellet was resuspended in DMEM/F12 medium. The cell number was counted for organoid fabrication.

#### 2.3.3 Fabrication and culture of NEOs and DEOs

To build NEOs, the primary tissues were mechanically dissociated, supplemented with or without enzymatic digestion of the extracellular matrix (ECM), to extract single cells and cell clusters (less than 15 cells). The extracts were suspended in Matrigel at 4 °C and pipetted into gel sheets or domes. The cell growth colonies were generated by incubating and prolonged culturing of the deposits at 37 °C in a defined medium [13,18]. After 2-week growth, small and inconsistent NEOs are obtained.

For the fabrication of DEOs, we adapted the protocol from Jiang et al.[17]. Briefly, primary cells were harvested and resuspended in growth factor-reduced Matrigel (Corning, US) with a density of ∼ 2 × 10^7^ cells/mL. The cell suspension and fluorocarbon oil (HFE-7000, 3M Novec) were loaded in a 1-mL syringe and a 10-mL syringe, respectively. The Matrigel phase and oil phase were injected into two polytetrafluoroethylene (PTFE) tubings (inner diameter: 560 µm, Cole-Parmer) with syringe pumps (TYD01-01-CE, Lead Fluid) in a 4 °C refrigerator. The tubings were connected to a hand-made polydimethylsiloxane (PDMS) 3-way connector, where the Matrigel phase was sheared into consistent plugs and flowed into another PTFE tubing. The Matrigel microspheres were incubated in the tubing at 37 °C for 15 min until fully solidified and then collected in PBS. Then the organoids were transferred into the corresponding culture medium (For mouse liver DEOs: basal medium DMEM/F12 (Thermo Fisher) supplemented with 20% fetal bovine serum, 1% penicillin-streptomycin, 100 ng/mL noggin (MCE), 100 ng/mL R-spondin 1 (MCE), 5 ng/ml EGF (Peprotech), 10 μM SB431542 (MCE), 2 μM CHIR99021 (MCE), 200 ng/mL FGF4 (MCE), 10 ng/mL FGF-basic (MCE), 5 μM Y-27632 (Abmole), and 1X FibrOut (Chi scientific). For human liver tumor DEOs: basal medium DMEM/F12 (Thermo Fisher) supplemented with 20% fetal bovine serum, 1% penicillin-streptomycin, 100 ng/mL noggin (MCE), 100 ng/mL R-spondin 1 (MCE), 5 ng/ml EGF (Peprotech), 10 ng/mL FGF-basic (MCE), 1X GlutaMAX Supplement (Thermo Fisher Scientific), 10mM HEPES (Thermo Fisher Scientific), 1X B-27 Supplement (Thermo Fisher Scientific), 5mM Nicotinamide (Sigma-Aldrich), 1.25 mM N-Acetylcysteine (Sigma-Aldrich), 5 μM Y-27632 (Abmole), and 1X FibrOut (Chi scientific)). The medium was changed every 3 days and DEOs were harvested on day 7.

#### 2.3.4 Fabrication and Culture of NEOs

We adapted Nicola Valeri's protocol [18] to fabricate traditional organoids. After human tumor processing, ∼5000 cells were seeded on 30 μL Matrigel in Petri dishes and solidified in a 37 °C and 5% CO_2_ cell culture incubator for 20 min. Then, the culturing medium which was the same as the DEO culture medium was added to individual culturing wells. The medium was changed every 3 days and NEOs were harvested on day 14.

#### 2.3.5 Histology

Tissue and organoids were fixed in 4.0% paraformaldehyde (PFA) (Beyotime) and frozen under -20 °C. Afterward, they were cut into 10 μm slices by a Thermo Scientific NX50 freezing microtome. The sections were performed following the standard HE staining protocol. Images were acquired on a Leica DM1000 inverted microscope.

#### 2.3.6 Immunofluorescence

After being cultured for 7 days, DEOs were fixed with 4.0% PFA and washed three times using PBS (1 ×). Then, the organoids were permeabilized with 0.5% Triton X-100 for 5 min at room temperature. After incubation in 5% bovine serum albumin (Sigma-Aldrich) for 1 h, the organoids were incubated with antibodies against E-cadherin (1:200, Abcam), Albumin (1:50, Huabio) at 4 °C overnight. After washing with PBS (1 ×), the organoids were further incubated with Alexa Fluor 488 conjugated secondary antibody (1:500, Abcam). Finally, the nuclei were stained with DAPI (1:1000, Beyotime). The images were captured by a Nikon A1R+ Laser scanning confocal microscopy.

#### 2.3.7 Protein expression analysis

Liver maturation proteins were determined with ELISA (Bioswap) and BCA kit (Beyotime). About 80 DEOs were collected on day 7 after washing with PBS. DEOs were homogenized to evaluate tissue Alb, Gsta2, and Pxr expression. Protein expressions were normalized with total protein expression determined by the BCA kit. Each sample was tested with two repeats and examined with PlateReader (Beckman) within 5 minutes after the reaction.

#### 2.3.8 Cell viability and imaging

The cell viability of human liver tumor DEO was evaluated on day 1 by using a double (LIVE/DEAD) staining kit (Yeasen, Shanghai, China) containing Calcein AM and PI. Samples were washed three times with PBS and immersed in PBS (1 mL) supplemented with Calcein AM (1 µL, 2 × 10^−3^ m) and PI (3 µL, 1.5 × 10^−3^ m) for 1 h, then, washed three times again with PBS before imaging under a fluorescent microscope (Nikon Eclipse Ts2r).

#### 2.3.9 RNA-seq analysis

Approximately 300–400 organoids were used for each RNA-seq analysis. The organoids were suspended in a medium and pipette-transferred to a centrifuge tube. Organoids were heavier than the medium and sedimented to the bottom tip. The supernatant was removed and organoids were resuspended in PBS by gentle petting, and preserved in an ice bath before sequencing performance. Total RNA was extracted from the tissues using TRIzol (Invitrogen, Carlsbad, CA, USA) according to manual instruction. Sequencing data was generated on the BGIseq500 platform (BGI-Shenzhen, China). The filtered clean reads were aligned to the reference genome GRCm38.p6 or GRCh38.p13 using Bowtie2 [19] according to species, then the gene expression level of each sample was calculated by RSEM(v1.2.12) [20]. In our analyses, the only protein-coding gene was considered. Then we removed the genes whose maximum TPM across all samples was less than 1. And log2(tpm+1) was used to calculate the gene expression level.

#### 2.3.10 Identification of tissue-specific genes

To identify liver-specific genes, we analyzed the RNA-seq data across 17 tissues from Li, B et al. [21]. In our analysis, a gene was considered a liver-specific gene if its expression value was six times higher of log2 fold than any other tissue.

#### 2.3.11 Assessment of cell-type proportions

We applied Scaden (https://scaden.readthedocs.io) to perform cellular deconvolution based on our bulk RNA-seq data [22]. For mouse liver tissue and liver-derived organoids, we used the single-cell transcriptome data from Tabula Muris (https://tabula-muris.ds.czbiohub.org) to simulate the training data. For liver cancer and cancer-derived organoids, we used scRNA data from GSE151530 as a training dataset.

#### 2.3.12 Quantification and Statistical Analysis

The projective area of DEOs and NEOs are quantified using Adobe Photoshop, ImageJ, and Prism GraphPad. In brief, organoids’ boundaries were extracted with the object selective tool of Adobe Photoshop, and then organoid projective areas were quantified with ImageJ. Projective areas were transferred into log scale and plotted into violin plots with Prism GraphPad. Welch's t-tests and ANOVA were performed in Prism GraphPad. Pearson's correlation was calculated by Pandas (v1.3.4). We used Seaborn (v0.11,2) to plot the cluster heatmap.

#### 2.3.13 ssGSEA analysis

To estimate expression activity levels of biological processes, we performed a single sample GSEA (ssGSEA) analysis using the GSEAPY python module[23], and the normalized enrichment score (NES) was used to indicate liver function activation levels.

## 3 Results and discussion

### 3.1 Multi-dimensional comparison between DEOs and NEOs

Droplet-engineered organoids (DEOs) were proposed to tackle the problem of non-engineered organoids (NEOs) being inconsistent and below-satisfactory in phenotypic recapitulation.

**Fig. 1:**
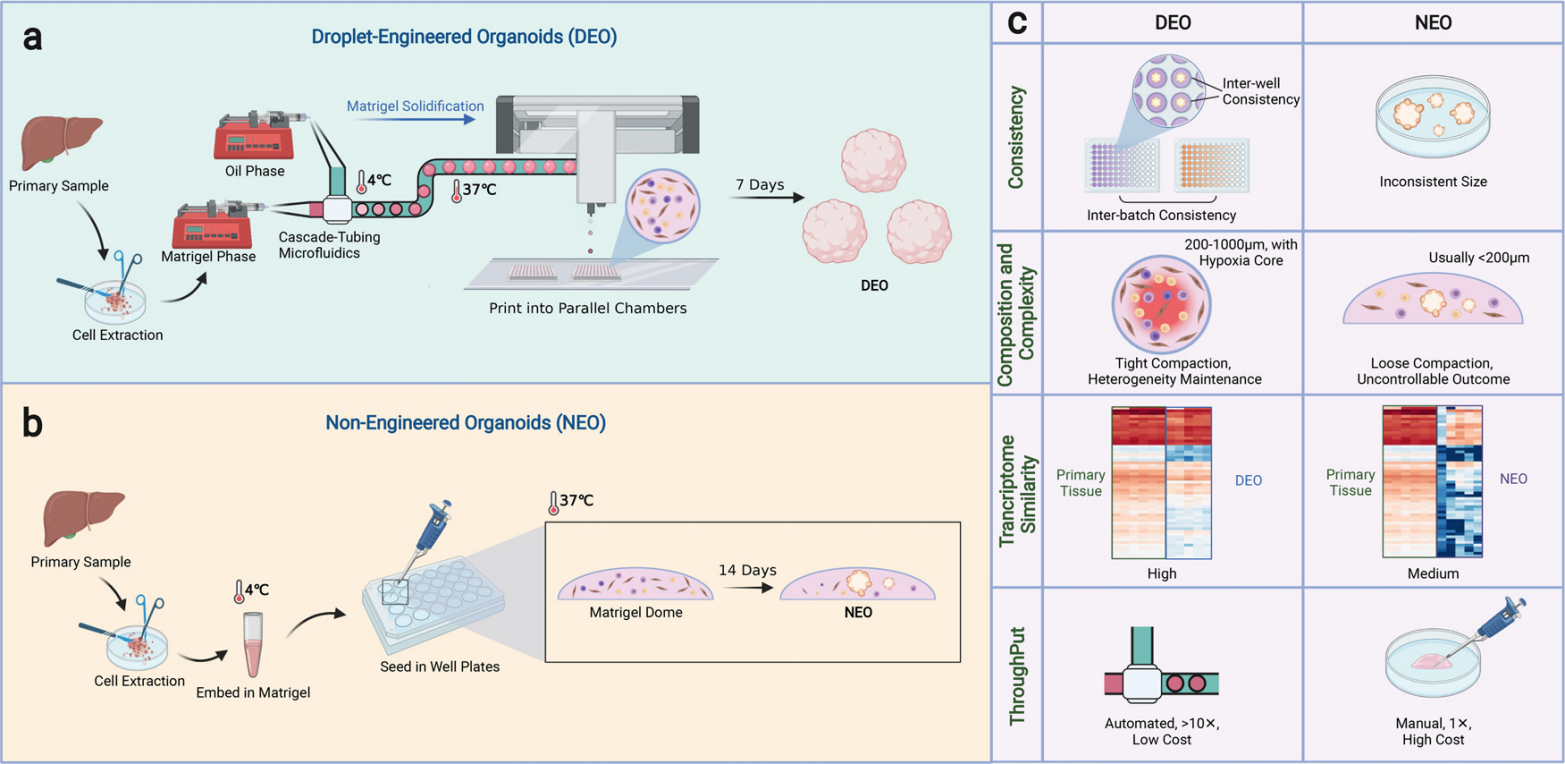
**Overview of the Droplet-Engineered Organoids (DEOs) and Non-Engineered Organoids (NEOs).** (a) Schematic showing the key steps to generate primary tissue (or tumor)-derived liver DEOs, by using a cascade tubing microfluidics (CTM) system synchronized with a printer for gel spheres. Organoids with high consistency are developed within 7 days. (b) Schematic showing the key steps to generate primary tissue (or tumor)-derived NEOs, by pipetting and gelling cell-laden Matrigel sheets or domes with heterogeneous cell colonies. Inconsistent organoids are developed in 14 days. (c) Properties of DEOs and NEOs.

For primary liver tissue (or tumor)-derived organoids, DEOs were produced by using microfluidics droplets as the homogeneous structural template, where cells grew and self-organized within the boundary formed by the solid-liquid interface. The cell-laden Matrigel droplets were formulated in a cascade tubing microfluidics (CTM) that had zero dead volume and minimal flow disturbance of droplet transconductance under surfactant-free conditions (Fig. 1a) [17,24]. Cell-laden Matrigel droplets were transferred into Matrigel beads, i.e. organoid precursors, during droplet conductance, extruded from the tubing, and collected. After 1-week culture, matured DEOs were characterized.

Non-engineered approaches are commonly used in laboratory studies because of their ease of use. NEOs were produced by manual pipetting of small volumes (usually 30 μL) of cell-in-Matrigel suspension, which formed a dome in plate wells and solidified after 15 min incubation at 37 °C. NEOs were obtained after 2-week growth with extremely high diversity in size (spanning tens to hundreds of microns) and shape (Fig. 1b).

The primary tissue-derived DEOs, compared with NEOs, show improved properties satisfying translational expectations in multiple aspects. First, DEO production and displacement are automated and highly consistent. The batch-to-batch variance is minimized (Fig. 1c, the top row). Within a single batch, the organoids are templated from monodisperse droplets comprising a defined population of cell mixture, thus the inter-organoid homogeneity (i.e. inter-well consistency) is guaranteed. The NEOs, however, are inconsistent due to the undefined cell colonies. Moreover, DEOs are advantageous in the recapitulation of tumor cell composition, complexity, and size tailorability. DEOs vary from 200 to 1000 μm as defined by the droplet sizes, whereas NEOs are generally less than 200 μm even after growing for 2 weeks (Fig. 1c, the middle row).

The improved complexity, microenvironment, production throughput and spectrum, and transcriptome profiling are attributed to the boundary condition decided initially by the liquid-oil interface of the templating droplets. In the defined volume, the cell distances are shortened, thus intercellular signaling and heterogeneous cell co-culturing are augmented. Cell consumption for an individual organoid colony is dramatically reduced because all compartmentalized cells (e.g. 1000 cells) in a droplet contribute to the growth of a single organoid (e.g. 400 μm in diameter). On the contrary, the organoid volume is larger, because the initial cell spatial distribution is arranged in reduced three-dimensional (3D) volume. Therefore, for a small sample, e.g. a biopsy piece from an advanced cancer patient, nearly 100 DEOs are producible, satisfying the screening demand of nearly 10 drugs (Fig. 1c, the bottom row).

### 3.2 DEOs with inter-organoid homogeneity and parental histology

CTM produces consistent spherical or oval DEOs ranging from 200 to 1000 μm, below 100 nL in volume. Shape and DEO dimensions can be precisely defined by the determination of the aqueous (cells in Matrigel)-to-oil flow rate ratio and the tubing inner diameter [25]. In this paper, we chose a modest configuration to produce slightly oval DEOs of ∼ 560 μm wide, for the templating droplets and the rendered organoid precursors.

**Fig. 2:**
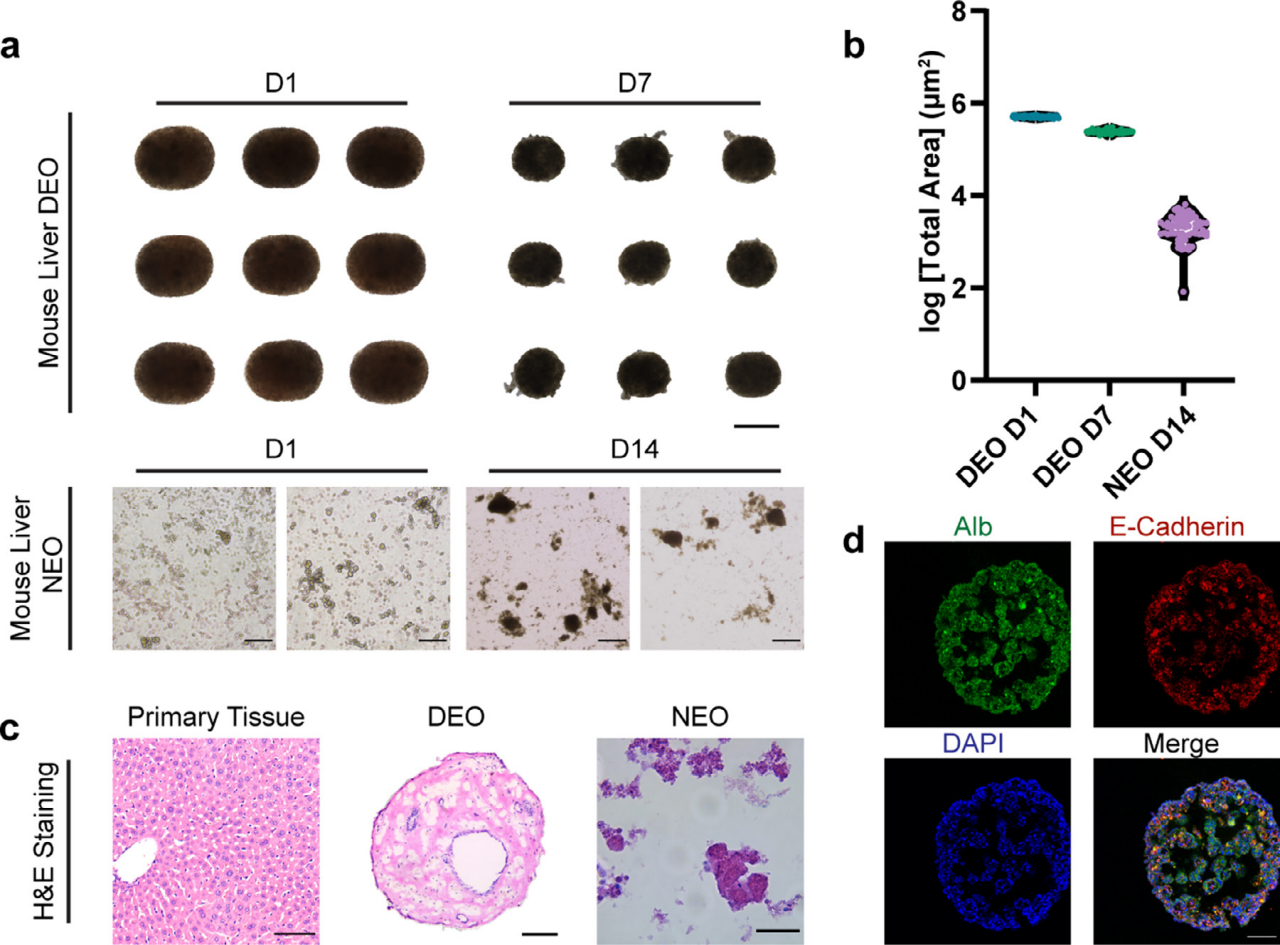
**DEOs derived from mouse liver tissues are highly reproducible and larger than NEOs.** (a) DEOs and NEOs derived from mouse liver tissues are grown for 1 day, 7 days, and 14 days, respectively after cell-laden Matrigel (2 × 10^7^cells per mL) droplets formulation or pipette deposition. Scale bars, top row, 500 μm; bottom row, 100 μm. (b) The violin plots of the cross-sectional area (projected area), in the log phase of DEOs (Day 1, Day 7) and NEOs (Day 14) derived from mouse liver tissues. (c) Mouse primary liver tissue, a derived-DEO, and a NEO with H&E staining. Scale bars, 100 μm. (d) A DEO derived from mouse liver tissue on Day 7 co-stained with Alb (green) E-Cadherin (red) and DAPI (blue) to illustrate the epithelium and cell nuclei, respectively. Scale bars, 100 μm.

Primary liver tissues were mechanically dissociated to extract single cells and small cell clusters (less than 15 cell aggregates). The mechanical dissociation already achieved high cell extraction efficiency from both liver tissues and tumors, in the absence of enzyme digestion. Minimal biochemical manipulations without further cell sorting were implemented to preserve cell viability and maintain heterogeneous cell populations. Mouse primary liver DEO precursors showed regular consistent morphology with an oval shape on day 1 (Fig. 2a, upper left). DEO precursors developed into organoids on day 7 with a slight ellipse shape (Fig. 2a, upper right**)**. Mature DEOs' diameters shrank to around 400 μm wide, which was probably because of collective tensile forces generated by cell–cell connection and cell-ECM connection. This was supported by the fact of reduced aspect ratio in the appearance of the reorganized shapes, i.e. matured DEOs. However, irregular cell outgrowth and unsmooth boundaries emerged, which were attributed to the growth in cell count and cell self-organization associated with hepatic feature development. Both DEO precursors and mature DEOs showed dense dark morphology because of high initial cell density, cell condensation by self-organization and remodeling on matrix, and, perhaps, cell proliferation.

Similarly, the mixture of single cells and cell clusters was embedded into Matrigel and deposited as sheets or domes to initiate organoid development for NEO production. Single liver cells and cell clusters were observed on day 1. NEOs were obtained after 2-week culture. They varied vastly in size, shape, and tissue density, as reflected by the tissue transparency under uniform illumination (Fig. 2a, bottom). Due to the random distribution and large span of initial cells, local cell composition and density varied. Uncontrolled initial parameters might lead to expanded uncertainty and challenge further translation and applications of NEOs. Consistent with DEOs, successful NEOs show dark, dense morphology, while the shape varied from sphere to polygon. Loosely packed cell clusters without small volume confinement led to growth failure (Fig. 2a, bottom right, black arrow).

Compared to NEOs, DEOs showed consistent initial and developed sizes. We quantified the projected area of DEOs (day 7) and their precursors (day 1), and NEOs (day 14). The polydispersity index (PDI) adapted from ref. [26] is evaluated with Eq. 1 and displayed on the logarithmic scale.

(1) $$\text{PDI}=\frac{\sqrt{\langle Area_{proj}^{2}\rangle -{\langle Area_{proj}\rangle }^{2}\;}}{\langle Area_{proj}\rangle }$$

DEOs possessed inter-organoid homogeneity (Fig. 2b). DEO precursors display monodisperse distribution with a PDI of 4.47%, which suggests the consistency and reliability of CTM. Cell reorganization led to morphology variety on day 7, with the PDI reaching 8.94%. The projected area data proved the shrinkage during the development of DEOs. NEOs showed a wide distribution of projected area. With the PDI of 55.96%, the NEOs morphology profiles were highly unstable. The projected areas of NEOs on day 14 were nearly 3 magnitudes smaller than DEOs on day 7. It suggested the limitations of NEOs, including smaller sizes, higher time cost, and perhaps, less complexity than DEOs.

DEOs recapitulated normal liver histology. Within a DEO, cells were organized with a typical epithelium structure. The large scale of the ordered and continuous structure indicated that there's a single large organoid derived from a Matrigel droplet template; instead of multiple, smaller, and inconsistent organoids in a single Matrigel sheet or dome from non-engineered approaches. The porous morphology of the DEO resembled its primary tissue. Some NEOs showed typical outgrowth hepatic structures, but others displayed loosely-packed amorphous morphology, reflecting the poor reproducibility of NEOs (Fig. 2c). The morphological difference between pure hepatic organoids and cholangiocyte organoids was attributed to the heterogeneity in the initial cell population and its maintenance. Whole organoid immunostaining showed a wide distribution of the epithelium adhesion marker, E-cadherin, and liver-specific marker, albumin. DEO recovered the typical liver organoid epithelium-dominant structure (Fig. 2d and Figs. S1 and S2). In summary, it suggested the advantages of DEOs over NEOs, including larger sizes, higher consistency, faster modeling, and increased complexity.

### 3.3 Higher transcriptomic correlation and consistency between DEOs and parental tissues

**Fig. 3:**
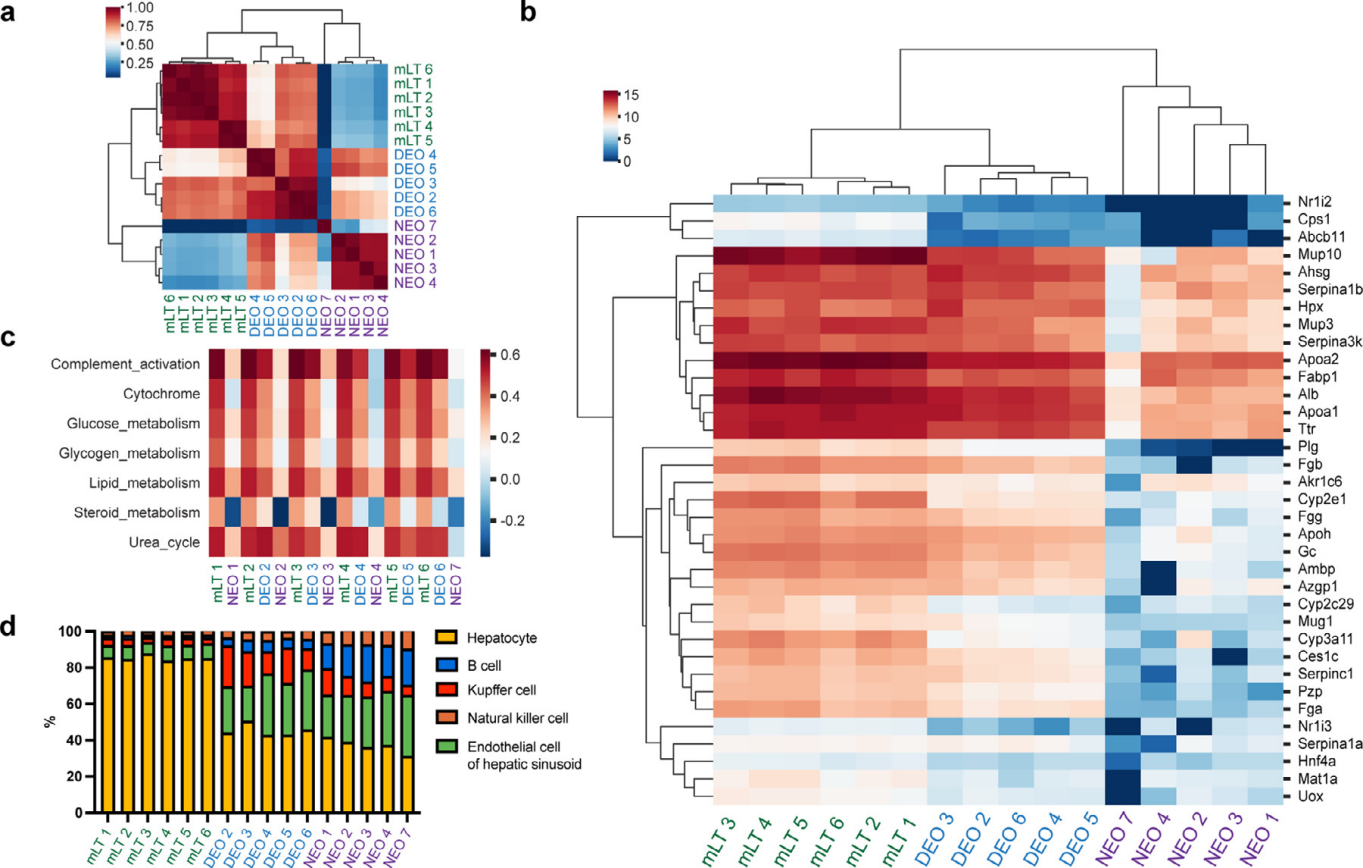
**Transcriptomic analysis of mouse liver tissues (mLTs) and the derived DEOs and NEOs by RNA-seq.** The DEOs and NEOs were collected for RNA-seq on day 7 and day 14, respectively. (a) The Pearson correlation cluster heatmap of mLTs, DEOs, and NEOs. The DEOs are highly correlated with each other and exhibit a higher correlation with their parental mLTs than the corresponding NEOs. The integers in labels index the samples. (b) The expression heat map of liver-related genes in mLTs, DEOs, and NEOs. The DEOs exhibit a higher correlation with their parental mLTs than the corresponding NEOs, which are also more heterogeneous. (c) The results of ssGSEA in mLTs, DEOs, and NEOs. Gene sets are primarily from Katsuda, T. *et al*[33]. For each sample, the DEO exhibits a closer expression profile with the parental mLT than the NEO. (d) Cellular deconvolution outcome of mLTs, DEOs, and NEOs from RNA-seq profiling.

To assess whether DEOs perform better than NEOs in recapitulating the expression profile of the corresponding parental tissues, bulk mRNA sequencing (RNA-seq) was performed for both organoids and their parental mouse liver tissues. Gene expression correlation analysis indicated that each DEO had a higher correlation to its parental tissue than its counterpart NEO. Hierarchical cluster analysis displayed that mouse liver tissues (mLTs) and DEOs were grouped into one cluster, but the NEOs formed another cluster. The obvious cluster separation of mLTs and DEOs from NEOs proved the efficient transcriptome restoration of the droplet-engineered methodology (Fig. 3a). The inter-tissue correlations were all above 0.88, which indicated the high consistency of the sampled mouse tissues and limited intra-species transcriptome diversity. The similarities of DEOs to their parental tissues were all above 0.62. Specifically, DEO 2, 3, and 6 (the batch indexes) showed higher similarity to their parental tissues, with Pearson correlation values over 0.79. On the contrary, the correlation between NEOs and their parental tissues was all less than 0.3.

Then we evaluated liver-specific gene expression patterns in liver tissues and the derived DEOs, NEOs. Genes highly expressed in the liver against other tissues were selected as liver-specific genes. The expression levels of selected genes were displayed on a log scale and showed similar patterns between the mLT and the DEO cohort in the cluster heatmap, but were distinguished from the NEO cohort (Fig. 3b). Liver markers such as plasma proteins (*Apoa1, Ahsg, Fga, Fgg*), enzymes (*Hao1, Rdh16*), hepatic markers (*Hpx, Tat, Ttr, Cyp3all*), and hepatocyte fate gene (*Serpina1b*), had higher expression levels in DEOs and the parental tissues than in NEOs. Moreover, each DEO batch presented a similar gene expression pattern, recapitulating the intra-species consistency of the sampled tissues, and confirming the inter-batch reproducibility of derived DEOs.

**Fig. 4:**
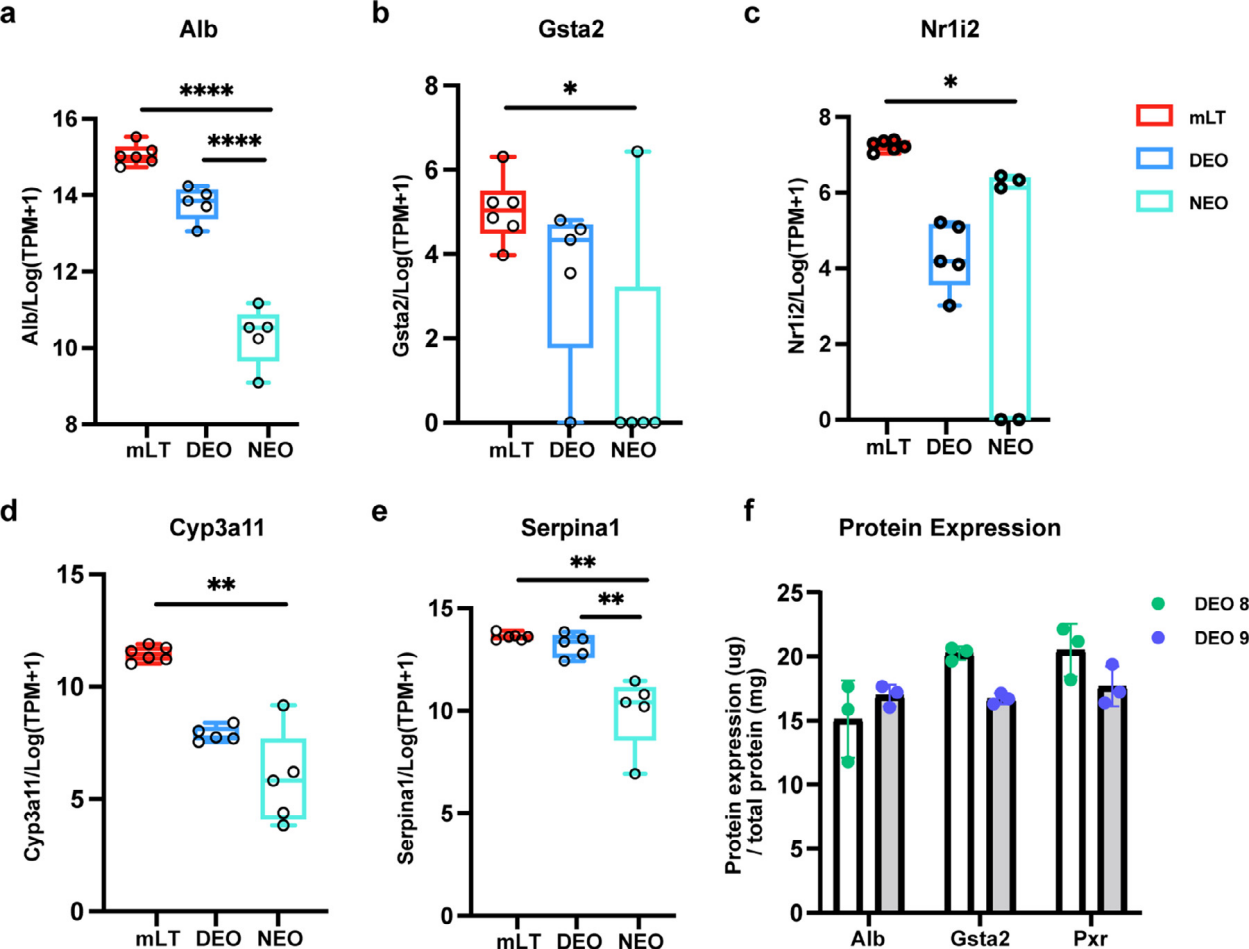
**Gene and protein expression of hepatic maturation markers.** Gene and protein expression of hepatic maturation markers. Gene expression levels including (a) *Alb*, (b) *Gsta2*, (3) *Nr1i2*, (d) *Cyp3a11* and (e) *Serpina1* are compared between DEOs, NEOs and parental tissues (mLTs). (f) DEO expression of liver-specific proteins is quantified by ELISA on day 7, including albumin (Alb), glutathione S-transferase alpha 2 (Gsta2), and pregnane X receptor (Nr1i2). Protein expression levels are normalized by the amount of total protein, determined by the Bicinchoninic Acid Assay (BCA). Alb, albumin; Gsta2, glutathione S-transferase alpha 2; Pxr, pregnane X receptor. Statistical significance within the different organoid techniques was first tested by ANOVA and specific pairwise comparisons were made using Welch's t-tests. Stars above each column indicate the statistical significance relative to the media condition. Bars with stars indicate statistically significant differences between the two indicated techniques. * *P* < 0.05, ** *P* ≤ 0.01, *** *P* ≤ 0.001, **** *P* ≤ 0.0001.

Next, we performed a single-sample Gene Set Enrichment Analysis (ssGSEA) to assess the activity levels of specified biological processes beyond single gene expressions [23]. Most liver function-related gene sets were upregulated in the liver tissues (mLTs) and liver-derived DEOs, such as the Cytochrome P450 activity, glycogen, metabolism, lipid metabolism, urea cycle, and complement activation (Fig. 3c), whereas only lipid metabolism displayed a similar expression level between NEOs and mLTs. Besides, we analyzed the expression level of liver-specific genes related to liver function. Compared with NEOs, DEOs had higher Cyp3a11, Serpina1d, and Alb expressions. Some hepatic maturation markers, including Gsta2 and Nr1i2, cannot be detected in NEOs (Fig. 4a-e)**.** Liver maturation protein expressions were determined by ELISA as well (Fig. 4f).

Finally, we applied a cellular deconvolution algorithm to calculate the cell proportions in mLTs, DEOs, and NEOs, based on their transcriptomic dataset (Fig. 3d). The results of deconvolution displayed similar cell types between DEOs, NEOs, and parental mLTs, suggesting that the major cell types in the mouse liver tissues were recapitulated in both DEO and NEO models. However, both DEOs and NEOs displayed significantly increased fractions of some cell types other than hepatocytes. DEOs recapitulated parental NK cell, and B cell populations better than NEOs, which might suggest tight compaction of immune cells in micro-droplet volumes helped maintain original cellular ratios. NEOs showed high similarity in the Kupffer cell population with primary tissues than DEOs. Neither DEOs nor NEOs recapitulated the endothelial cell population, probably because of the lack of the proper condition for endothelium growth. Further validations were required for absolute composition determination. For example, the high-cost single-cell RNA-seq technique may present a more delicate comparison that helps to decipher cues for organoid growth regulation.

In summary, by comparing the expression levels *from both single genes and grouped gene sets and the cell compositions,* a conclusion was reached that, for normal liver tissues, DEOs possessed improved inter-organoid homogeneity, inter-batch consistency, and organoid-tissue consistency when compared with NEOs.

### 3.4 Human liver tumor-derived DEOs recapitulate inter-tumor heterogeneity

Human tumors have strong heterogeneity that results in poor overall therapeutic significance. Recapitulating tumor heterogeneity becomes a major goal, and meanwhile, a challenge, for tumor modeling *in vitro*. To validate DEO as a versatile and more efficient modeling tool not only for normal primary tissue but for human tumors, we performed transcriptomic analysis for both DEOs and NEOs, derived from both HCC (Hepatocellular Carcinoma) and CC (Cholangiocarcinoma) patient tumors.

**Fig. 5:**
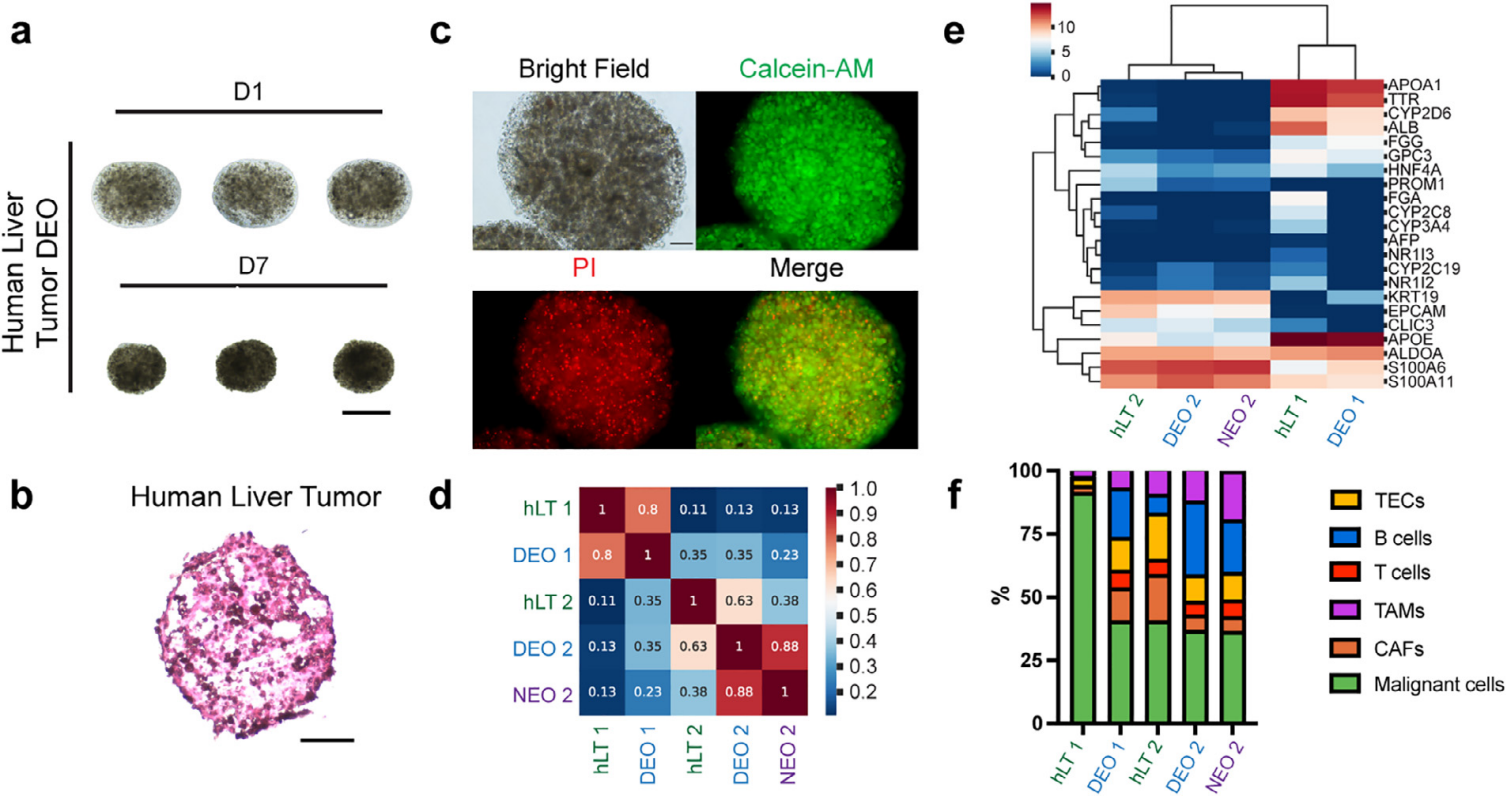
**Characteristics of human liver tumors (hLTs) and the derived DEOs and NEOs.** (a) DEOs derived from hLTs on Day 1 and Day 7. Scale bar, 500 μm. (b) A DEO derived from hLT with H&E staining on Day 7. Scale bar, 100 μm. (c) A human liver tumor-derived DEO on day 7, stained with Calcein-AM and PI to illustrate the live and dead cells, respectively. Scale bars, 500 μm. (d) The heat map of Pearson correlation coefficients of hLTs, DEOs, and NEOs. The DEOs exhibit a higher correlation with their parental mLTs than the corresponding NEOs. (e) The expression heat map of cancer-related genes in hLTs, DEOs, and NEOs. The DEO exhibits a closer expression profile with the parental mLT than the NEO. (f) The cellular deconvolution outcome showing the composition of hLTs, DEOs, and NEO.

The implementation using CTM to fabricate liver tumor DEOs followed the similar principles as for mouse liver tissues. Similar to mouse liver DEOs, tumor DEOs (day 7) exhibited obvious volume shrinkage in culture compared with their precursors (day 1). But both precursors and developed DEOs maintained inter-organoid homogeneity, judging from their consistent shape, size, and cell density (Fig. 5a). The H&E staining showed that tumor DEOs are developed as an integral and retain tumor morphology (Fig. 5b)**.** LIVE/DEAD staining presented abundant viable cells homogeneously distributed in the droplet-templated volume (Fig. 5c and Video S1). In-droplet shear force in CTM tended to cause minimal impact on initial cell viability.

Next, we quantified and compared gene expression profiles of the whole transcriptome, liver cancer-specific genes, and cellular spectrum in human liver tumors (hLTs), DEOs, and NEO, to verify the superior efficacy of DEOs over the counterpart NEOs. For the two tumor samples, HCC and CC, each organoid group correlated to its parental tumor, but distinguished significantly from the other tumor subtype (Fig. 5d)**.** The whole transcriptome correlation heatmap of the CC-derived DEO and NEO with the parental CC tumor proved that the DEO has a higher capacity to recapitulate the tumor transcription profile.

Next, we compared a cohort of liver cancer-specific markers to assess the DEOs' gene expression profiles (Fig. 5e). The HCC marker (*GPC3)* and hepatocyte markers (*ALB, TTR, APOA1, APOE*) were highly co-expressed in both DEO1 and hLT1. In contrast, CC markers (*EPCAM, KRT19*) were highly expressed in DEO2 and hLT2. DEOs could preserve the tumor transcriptional heterogeneity in different tumor subtypes.

Finally, we applied a cellular deconvolution algorithm on bulk RNA-seq data to obtain cell-type proportions in parental tumors and derived organoids (Fig. 5f). The results of cell-type deconvolution indicated that both DEO and NEO modeling could recapitulate major cell compositions in liver tumors; however, the proportions of cell types other than malignant cells were unable to exactly replicate the scenarios in tumors.

In summary, DEOs demonstrated promising properties for translational applications in many aspects. Compared with NEOs, DEOs possess inter-organoid homogeneity and inter-tumor cell heterogeneity, and significantly higher similarity to parental tissues both in cell composition and transcriptome profile (Table 1). Moreover, it costs less time to construct DEOs (e.g. 1 week) than NEOs (e.g. 2 weeks). The high tailorability of DEOs' sizes (200–1000 μm) within a definite timeframe provides more opportunities for translational uses.

**Table 1:** **Properties of DEOs and NEOs**.

	Source of Materials	Modelling Time	Size*	Dispersity#	Cellular Spectrum Recapitulation+	Transcriptome Recapitulation+
DEO	Primary Tissue/Patient-derived	∼1 week	200-1000μm	<10%	High	High
NEO	Primary Tissue/Patient-derived	>2 weeks	<200μm	>50%	Medium	Medium

⁎ The equivalent diameter for the cross-sectional area

# Refers to PDI (see Methods)

+ Compared with the parental liver tissues or tumors

## 4 Conclusion

This study compares organoid products by using two different methods. The differences are attributed to the structural definition, with or without the boundary-conditioned spatial organization of a heterogeneous cell population. Though DEOs are proven superior to NEOs in the transcriptional recapitulation of the parental tissues or tumors, they need further improvement. For example, the cell composition is not entirely matching the parental tissue, especially in the fractions of individual cell types. Overall, the transcription similarity of DEOs against the parental tissues is not reaching 0.9 in most cases. However, the correlation values are significantly higher than the reported values of iPSCs-derived organoids [27].

The organoid technology intrinsically is invasive, requiring cell extraction, and re-packing of cells and their microenvironment. In this process, stimulating factors that are analogous to traumas are introduced, thus possibly causing inflammatory-alike responses, and augmenting immune activities. It might explain the elevated fractions of immune cells in the deconvolution.

Second, the modeling of healthy liver tissues is highly consistent, but the tumor modeling has lower consistency, as the two liver tumors demonstrated hepatocellular carcinoma and cholangiocarcinoma were proven with distinct transcription features. Moreover, the tumor and tumor organoid pairs are less similar, as quantified by the Pearson correlation coefficients, though the DEOs prove better recovery than their NEO counterparts. The lowered coefficients may be attributed to the instability of the tumor genome and transcriptome.

Droplet templating enables sub-microliter volume manipulation, and the spherical configuration shortens the cell-to-cell distance as in sheet- or dome-gel. Droplet templating augments cell signaling and recovers the cell composition by droplet cell encapsulation. The in-droplet (or in-sphere) cell growth may overcome the low recovery rate of non-small cell lung cancer in organoid modeling [8]. The forced co-culture of heterogeneous cell populations in boundary-confined submicroliter spheres may limit the overwhelming growth of unwanted (healthy) cells and maintain the structural and composition integrity among individual organoids [28]. In addition, to tailoring the spatial arrangement of encapsulated cells, the droplet templating effect also manipulates cells by controlling their extrinsic mechanical cues. Recently, mechanotransduction [29] has been proven of critical importance, and further improvements for DEOs are on the way following the reported elaboration. Cell signaling and mechanotransduction with both materials volume properties [30,31] and topographical cues [32] will be studied in the future.

## Materials availability

This study did not generate new unique reagents.

## Data and code availability

The data and codes for the reproduction of the results are available at a GitHub repository (https://github.com/heng-fa/Evaluate_NEO).

## Declaration of competing interest

The authors declare that they have no conflicts of interest in this work.

## Appendix Supplementary materials

Image, video 1

Image, application 2
