# Supplementary material for: Droplet-engineered organoids recapitulate parental tissue transcriptome with inter-organoid homogeneity and inter-tumor cell heterogeneity
Source: Fundam Res. 2022 Jun 3;4(6):1506–14. doi: 10.1016/j.fmre.2022.05.018 (PMC11670719; doi:10.1016/j.fmre.2022.05.018)
Supplement: Supplementary file 2 [file mmc2.docx]

# *Supplementary Information*

# Droplet-engineered organoids recapitulate parental tissue transcriptome with inter-organoid homogeneity and inter-tumor cell heterogeneity

Haoran Zhao^a,b,1^, Yifan Cheng^a,b,1^, Jiawei Li^a,b,1^, Jiaqi Zhou^a,b^, Haowei Yang^a,b^, Feng Yu^a,b^, Feihong Yu^a^, Davit Khutsishvili^a,b^, Zitian Wang^a,b^, Shengwei Jiang^a,b^, Kaixin Tan^c^, Yi Kuang^c,d^, Xinhui Xing^a^, Shaohua Ma^a,b,^*


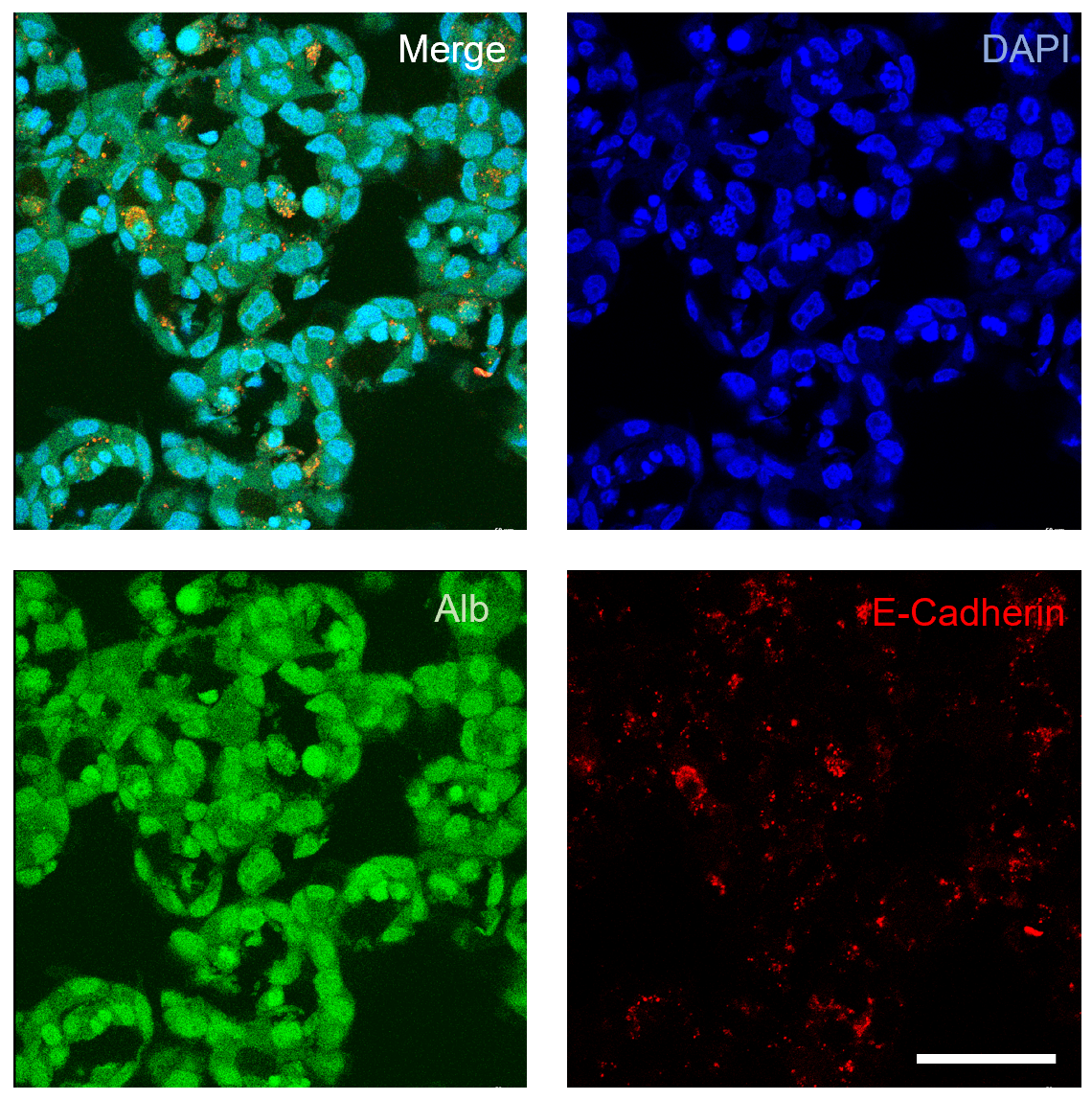


**Fig. S1. Immunofluorescence images of DEO at a higher magnification.** Scale bar: 50 μm.


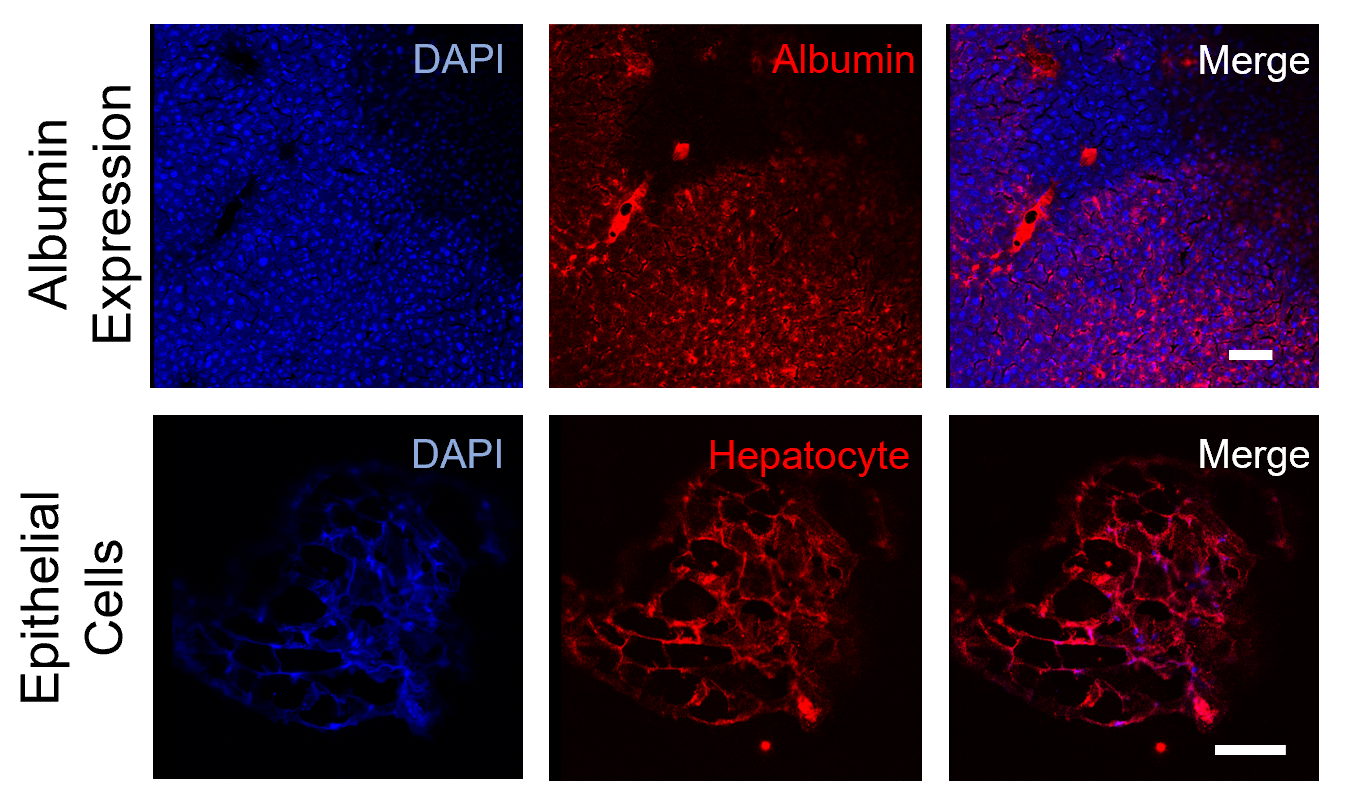


**Fig. S2. Immunofluorescence images of mouse liver tissue.** Scale bar: 50 μm.

**Video S1: Cell viability of a human liver tumor DEO at day 7.**
